# The Gut Microbiome and Schizophrenia: The Current State of the Field and Clinical Applications

**DOI:** 10.3389/fpsyt.2020.00156

**Published:** 2020-03-12

**Authors:** Tomasz Szeligowski, Alexandra Lim Yun, Belinda R. Lennox, Philip W. J. Burnet

**Affiliations:** ^1^St. Edmund Hall College, University of Oxford, Oxford, United Kingdom; ^2^Department of Psychiatry, University of Oxford, Oxford, United Kingdom

**Keywords:** inflammation, microbial communities, psychosis, antipsychotics, supplementation

## Abstract

Schizophrenia is a debilitating psychiatric disorder, leading to both physical and social morbidity. Despite its importance, the etiology of schizophrenia remains poorly understood. Furthermore, its mainstream treatments fail to address all aspects of the disorder and are associated with significant side-effects. Recently, there has been growing interest in the relationship between the gut microbiome and mental health, including in schizophrenia. In this article, we review the existing evidence implicating dysbiosis in schizophrenia and discuss how the presumed dysbiosis could fit within known hypotheses of its pathogenesis, focusing on inflammation, tryptophan metabolites, and BDNF levels. We also evaluate the clinical potential of manipulating the gut microbiome with probiotics and prebiotics as adjunctive treatments in schizophrenia, based on existing clinical and pre-clinical studies. Overall, the current data showing microbiome alterations in schizophrenia are highly discrepant and insufficient to conclude whether microbiome changes are associated with increased risk of the disorder, or are simply the result of external factors or treatment. Despite some encouraging results of pro/prebiotic supplementation, there is also inconclusive evidence for their efficacy in schizophrenia. Thus, further research and more clinical trials are needed to test the validity of manipulating the gut microbiome to improve the treatment of this disorder.

## Introduction

Schizophrenia is a debilitating psychiatric disorder, characterized by positive symptoms (delusions, hallucinations, aberrant flow of thoughts), and negative symptoms (apathy, withdrawal, slowness) (1). It affects ~21 million people worldwide, and causes significant physical and social morbidity (2, 3). Its heritability is estimated at ~80% based on concordance rates in monozygotic twins (4), while risk profile scores constructed from alleles associated with schizophrenia explain ~7% of variation in the liability to schizophrenia (5). Thus, genetics cannot fully explain its etiology, and so other factors, particularly the environment, need to be explored (6, 7). Furthermore, the current treatments for schizophrenia do not address all aspects of this disorder (8) and are associated with considerable side-effects (9), necessitating development of improved treatment strategies.

The gut microbiome has been implicated in psychiatric disorders, and is well-described for depression, with dysbiosis suggested in clinical studies (10) and fecal transplants from depressed patients to germ-free rats shown to induce depressive-like behaviors (11). The gut microbiome is a rich community of microbes which are diverse and personalized, though dominated by phyla *Bacteroidetes* and *Firmicutes* (12). It is also dynamic, influenced by lifestyle factors such as diet (13), sleep (14), or stress (15). The personal and dynamic nature of the gut microbiome is why dysbiosis remains a poorly defined concept, despite the common use of this term in the literature. Accurate definition of a healthy baseline microbiome is near impossible to establish, so it is unclear what qualitative and quantitative changes in the microbiome composition constitute functionally significant deviations from the “norm,” what thresholds should be set when assessing the importance of relative deviations from controls, and whether these should be different for various populations. Bearing these limitations in mind, we take the term dysbiosis to mean microbiome profiles which are significantly different from controls and which could have functional significance in pathological processes if conclusively proven. Research into the role of the microbiome in schizophrenia is still in its infancy. In this article, the evidence implicating dysbiosis in schizophrenia, and how it could fit within existing hypotheses of schizophrenia pathogenesis will be appraised. Furthermore, the therapeutic potential of pre/probiotics in schizophrenia will be discussed.

## Evidence for Dysbiosis in Schizophrenia

To date, six studies have been published investigating microbiome differences between healthy controls and individuals with schizophrenia (Table 1). Unaltered microbial richness/diversity was predominantly reported, but with significant differences in the abundance of specific taxa between groups. Interestingly, one study reported that among patients who clustered with controls at baseline, 70% experienced remission within 12 months, compared to only 28% with “abnormal” microbiota—an association which was not weakened by including baseline GAF score as a covariate (21). There is, however, considerable discord between these results. At phylum level, *Proteobacteria* and *Firmicutes* were found both significantly elevated and reduced in schizophrenia. This is also the case for taxa within the class *Clostridia*, despite one study identifying this class as a whole to be enriched in schizophrenia (19). Importantly, one of these studies investigated the oropharyngeal microbiome which is structurally distinct from the gut microbiome (20). Perhaps the only truly consistent finding is a significant elevation of *Lactobacilli* in schizophrenia and people at increased risk of schizophrenia, which even correlated with symptom severity (21). This is perplexing, considering that *Lactobacilli* are common components of probiotics, thought to confer benefits for mental health.

**Table 1:** Summary of studies implicating dysbiosis in schizophrenia.

**Study design**	**(Microbial richness/diversity)**	**Significantly more abundant taxa in schizophrenia**		**Significantly more abundant taxa in controls**	
^a^**Study group**: 81 high-risk subjects (at least one first-degree relative with schizophrenia), 19 ultra-high risk subjects (defined by structured interview for prodromal syndromes), 69 healthy controls; all participants were 13–30 year-old Han Chinese individuals **Exclusion criteria**: GI and endocrine diseases, serious organ disorders, history of psychiatric disorders and corresponding treatment, use of medication within previous 3 months **Experimental method**: 16S rRNA gene sequencing from fecal samples	No significant difference between the three groups	**Orders:** *Clostridiales, Lactobacillales, Bacteroidales* **Genera:** *Lactobacillus, Prevotella* **Species:** *Lactobacillus ruminis*	Not reported		
^b^**Study group**: 64 schizophrenic patients, and 53 healthy controls; 18–65 year-old Han Chinese individuals **Exclusion criteria**: chronic disease in the last 3 months, use of medication within the last 6 months (including antibiotics and probiotics, but excluding antipsychotic medication) **Experimental method**: 16S rRNA gene sequencing from fecal samples	No significant difference	**Phyla:** P*roteobacteria* **Classes:** *Gammaproteobacteria* **Orders:** *Aeromonadales, Fusobacteriales* **Families:** *Veillonellaceae, Prevotellaceae, Enterobacteriaceae, Succinivibrionaceae, Fusobacteriaceae, Lactobacillaceae*	**Genera:** *Succinivibrio, Megasphaera, Collinsella, Clostridium, Klebsiella, prevotella, Lactobacillus, Fusobacterium, Citrobacter, Acidaminococcus, Desulfovibrio, Phascolarctobacterium* **Species:** *Collinsella aerofaciens, Bacteroides fragilis, Prevotella stercorea, Lactobacillus mucosae, Bifidobacterium adolescentis*	**Phyla:** *Firmicutes* **Classes:** *Clostridia* **Orders:** *Clostridiales* **Families:** *Lachnospiraceae*, Alkaligenaceae **Genera:** *Blautia, Coprococcus, Roseburia, Streptococcus*	**Species:** *Roseburia faecis, Blautia producta, Collinsella plebeius, Bacteroides eggerthii*
^c^**Study group**: 63 schizophrenic patients, 69 healthy controls; most patients receiving antipsychotic medication; participants recruited from the First Affiliated Hospital of Chongqing Medical University **Exclusion criteria**: other physical and mental disorders; use of antibiotics, probiotics, and prebiotics within 1 months before sample collection **Experimental method**: 16S rRNA gene sequencing from fecal samples	Lower indices of both microbial richness and diversity in schizophrenic patients	**Families:** *Veillonellaceae, Prevotellaceae, Bacteroidaceae, Coriobacteriaceae* **Genera:** *Megasphera, Akkermansia, Fusobacterium, Prevotella*		**Families:** *Lachnospiraceae, Ruminococcaceae, Enterobacteriaceae, Acidaminococcaceae, Rikenellaceae*	**Genera:** *Citrobacter, Blautia, Coprococcus, Lachnoclostridium* **Species:** *Bacteroides eggerthii, Bacteroides massiliensis, Collinsella stercoris*, *Haemophilus parainfluenzae*
^d^**Study group**: 25 outpatients with schizophrenia or schizoaffective disorder, 25 healthy controls; aged between 30–76; most schizophrenic patients were receiving antipsychotic medication **Exclusion criteria**: Other major psychiatric disorders, substance abuse, major neurological disorders **Experimental method**: 16S rRNA gene sequencing from fecal samples	No significant difference	**Classes**: *Clostridia, Erysipelotrichi* **Orders:** *Clostridiales* **Genera:** *Anaerococcus, Haemophilus, Sutterella, Megasphera, Coprococcus, Blautia*, *Ruminococcus*		**Phyla:** *Proteobacteria* **Genera:** *Haemophilus, Sutterella* **Species:** *Haemophilus parainfluenzae*	
^e^**Study group**: 16 schizophrenia patients and 16 controls; age 18-65; recruited from the Stanley Research Program **Exclusion criteria**: substance abuse, mental retardation, HIV infection, disorders affecting cognitive functioning **Experimental method**: Illumina sequencing of DNA extracted from throat swabs	Lower microbial richness in schizophrenic patients	**Phyla:** *Firmicutes* **Genera:** *Bifidobacterium, Lactobacillus* **Species:** *Lactobacillus gasseri, Catenibacterium mitsuokai, Lactobacillus salivarius, Bifidobacterium pseudocatenulatum, Streptococcus gordonii, Streptococcus thermophiles*		**Phyla:** *Bacteroidetes, Actinobacteria*	
^f^**Study group**: 28 first psychotic episode patients and 16 controls from Finland; most patients were receiving antipsychotic medication for a median of 20 days **Exclusion criteria**: substance-induced psychosis and psychosis due to a general medical conditions **Experimental method**: 16S rRNA gene sequencing from fecal samples	Not reported	**Families:** *Lactobacillaceae, Halothiobacillaceae, Brucellaceae, Micrococcineae* **Genera:** *Lactobacillus, Bifidobacteria, Tropheryma, Halothiobacillus, Saccharophagus, Ochrobactrum, Deferribacter, Halorubrum*		**Families:** *Veillonellaceae* **Genera:** *Bacteroides, Ruminococcaceae, Gallionella, Anabaena, Nitrosospira*	

a *He et al. (16)*.

b *Shen et al. (17)*.

c *Zheng et al. (18)*.

d *Nguyen et al. (19)*.

e *Castro-Nallar et al. (20)*.

f *Schwarz et al. (21)*.

Several factors could explain these inconsistencies. Firstly, all studies used small sample sizes (<100 patients), which could explain why certain differences did not reach statistical significance, and why differences are reported at selected taxonomic levels, thus hindering comparisons between studies. Reporting differences at selected taxonomic levels has an additional pitfall—members of the same taxon may differ significantly in terms of their physiological role and impact on pathological processes. This is obviously true for higher taxonomic levels which include increasingly dissimilar groups of bacteria, but is likely also true at genus or even species level. Such variation could explain why *Lactobacilli* can be implicated in both the pathogenesis of psychiatric disorders and confer benefits in them, depending on which specific subtype of is present. Secondly, the studies differed in exclusion of potential confounders, such as smoking, or co-morbid metabolic and cardiovascular illnesses, all of which affect the microbiome. Patient groups also differed in age, stage of illness, and treatment status. Indeed, antipsychotics have significant anti-commensal activity (22), and in rodents, olanzapine has been shown to elevate *Firmicutes* and decrease *Bacteroidetes* (23, 24), which was paralleled in human antipsychotic trials (25, 26), although not universally (27). These results bear resemblance to some of those reported in Table 1, and suggest that the microbiome was affected by treatment status. However, one implication of this is that if antipsychotic treatment impairs the microbiome, then auxiliary interventions aimed at restoring microbiome function could prove beneficial. Thirdly, the methods used in these papers are not consistent, which might alter the obtained taxonomic profiles and further hinder comparisons. While most studies utilized 16S rRNA gene sequencing as the taxonomic marker, different regions of the gene were used (V3 and V4) along with different primers, which is known to affect the outcomes of 16S rRNA gene-based diversity studies due to variation in the evolutionary rates of different regions of this gene (28). Additionally, one study used shotgun metagenomic sequencing on throat swab samples (20). Aside from the problem of differences between oropharyngeal and intestinal microbiomes, an important caveat of this approach is that the abundance of host DNA in swab samples may impair the accuracy of microbiome profiling (29). Considering the intrinsic variations in microbiome profiles, methodological consistency is necessary to generate data which will allow useful and accurate comparisons across studies.

The existing studies implicating dysbiosis in schizophrenia are all cross-sectional, hence causal relations cannot be inferred. Thus, large-scale prospective studies are needed, to identify whether certain microbiome profiles are associated with increased risk of developing schizophrenia. Fecal microbiome transplants have also been used to demonstrate causal roles of microbiome in disease, but will be challenging to implement in schizophrenia due to the lack of accurate animal behavioral correlates. In one study, fecal transplantation from schizophrenia patients to GF mice led to hyperactivity, reduced immobility in the forced swimming test (behavioral despair), and exaggerated startle response (18). Although these behaviors are seen in schizophrenia, they are not unique to it, and certain tests such as the Y-maze, sociability test, and pre-pulse inhibition test did not reveal differences, suggesting either that the microbiome does not affect all aspects of schizophrenia, or that the findings were not actually indicative of a schizophrenia-like phenotype. Finally, there is very little data on possible sources of dysbiosis, with C-section—a commonly suggested cause of dysbiosis (30)—found not to be associated with schizophrenia (31, 32).

## Potential Diagnostic Application

Two key studies have explored whether microbiome differences could serve as biomarkers for schizophrenia. One investigation demonstrated that the disorder is associated with changes in *Gammaproteobacteria* at class level, *Enterobacteriales* at order level, and *Bacteroides fragilis* at species level (17), whereas another determined that a panel consisting of *Aerococcaceae, Bifidobacteriaceae, Brucellaceae, Pasteurellaceae*, and *Rikenellaceae* is sufficient to distinguish patients from controls (18). However, much like with data in Table 1, there is discord in the bacterial taxa identified as markers in these studies, which could again reflect small sample sizes and insufficient overlap between studied populations. Furthermore, investigations of the gut microbiome in depression identified similarly discordant patterns of alterations, and showed a degree of overlap to changes seen in schizophrenia (33). This lack of specificity limits the potential diagnostic usefulness of the data and indeed, its robustness in demonstrating dysbiosis that is characteristic to schizophrenia.

## How Could Dysbiosis Contribute to Schizophrenia?

Inflammatory responses have long been implicated in schizophrenia [reviewed in (34)], although their origins are not understood. Schizophrenia is associated with elevated levels of IL-6, IL-8, and TNF-α, and reductions in the anti-inflammatory IL-10 (35). Moreover, elevated antibodies to *Saccharomyces cerevisiae* (markers of intestinal inflammation) have been identified (36), which is consistent with GI pathologies being among the most common co-morbidities of schizophrenia (37). Dysbiosis could exacerbate inflammation in the disorder through increased intestinal permeability. Indeed, elevated levels of the bacterial translocation marker sCD14 have been observed in schizophrenia (38), and in another study *Roseburia, Coprococcus*, and *Blautia* were reduced (17). These species mediate butyrate production (39), which has been shown to enhance the intestinal barrier function by facilitating tight junction assembly in monolayers intestinal cell-lines (40). In addition, genes involved in immunity are among the most commonly appearing in GWAS analyses of schizophrenia (5), suggesting that perhaps a genetic component driving inflammation is more significant. It is also possible that dysbiosis facilitates bacterial infections and contributes to inflammation—a hypothesis consistent with a study indicating bacterial infections as risk factors for schizophrenia (41). Additionally, the gut microbiome has been reported to regulate blood-brain barrier permeability (42), and so dysbiosis could potentially facilitate CNS infection and inflammation, which are also associated with schizophrenia (43).

There also exists a link between the immune system and the conversion of tryptophan to kynurenate (44). Kynurenate is a broad-spectrum glutamate receptor antagonist, and N-Methyl-D-Aspartate Receptor (NMDAR) hypofunction is implicated in schizophrenia [reviewed in (45)]. Elevated kynurenate levels have been observed in post-mortem schizophrenia brain tissue (46), while rats treated with kynurenine-3-monooxygenase inhibitors have reduced pre-pulse inhibition (reflecting the inability of schizophrenia patients to filter out extraneous stimuli), along with increased firing rate and burst firing activity of VTA dopaminergic neurons, indicating the dopamine hyperactivity associated with schizophrenia (47). Indoleamine-2,3-dioxygenase (IDO) is responsible for the rate-limiting step in conversion of tryptophan down the kynurenine pathway. Its key function is depleting tryptophan during immune responses to prevent pathogenic growth and dampen immune responses (44). Hence, IDO is upregulated by cytokines such as IFN-γ and TNF-α (44), and in schizophrenia, increased TNF-α has been observed.

The gut microbiome might affect the balance of tryptophan metabolism directly. A study comparing germ-free to control mice identified that GF mice had significantly higher tryptophan availability and decreased kynurenine:tryptophan ratio, which was increased upon colonization (48). In another study, administration of *Bifidobacterium infantis* to rats elevated plasma kynurenate (49). However, a decrease in the kynurenine:tryptophan ratio was observed, indicating reduced overall tryptophan metabolism down the kynurenine pathway. Importantly, there are no studies directly linking specific microbiome profiles in schizophrenia to kynurenate alterations. It is also important to mention that quinolinic acid is an NMDAR agonist, so it is difficult to extrapolate alterations of the kynurenine pathway directly to NMDAR dysfunction.

## The Microbiome and Brain-Derived-Neurotrophic Factor (BDNF)

BDNF is a key neurotrophin involved in neurodevelopment, particularly in learning and memory processes, and neurodevelopmental models of schizophrenia often include BDNF alterations, focusing on its role in the cognitive dysfunction in the illness [reviewed in (50)]. Reduced BDNF levels have been observed both in post-mortem hippocampal samples (51), and in the plasma of drug-naïve patients with schizophrenia (52, 53), while low baseline BDNF levels are associated with worse response to antipsychotic treatment (54).

In rodent studies, broad-spectrum antimicrobials have been found to significantly lower BDNF and TrkB expression in mouse hippocampus (55), though another study with similar design found significantly increased BDNF levels in the hippocampus, paralleled by increased abundance of *Lactobacilli* and *Actinobacteria*, and decreased abundance of γ*-Proteobacteria* and *Bacteroidetes* (56). These conflicting results are problematic, and it remains unclear whether the BDNF changes were mediated by the microbiome and/or the antibiotics themselves. GF mice were found to have lower BDNF expression in the hippocampus in two separate studies (57, 58), however colonization of GF NIH Swiss mice with BALB/c microbiota was found to reduce BDNF expression at 1 week post-transfer compared to NIH Swiss microbiota, but not at 3 weeks (56). Again, none of these studies is directly related to the microbiome in schizophrenia, and more convincing data could be obtained from studies analyzing the effects of FMT from schizophrenia patients on BDNF expression. Interestingly, inflammatory responses have also been associated with decreased hippocampal BDNF expression, providing an additional potential link between BDNF levels and altered gut microbiome (59).

## Prebiotics in Schizophrenia

Prebiotics are substrates utilized by host microorganisms, providing favorable conditions for “beneficial” bacteria (60). They commonly include non-digestible fructan oligosaccharides (FOS) and galactan oligosaccharides (GOS), selectively degraded by *Bifidobacteria*. Recently, a study has shown the potential of using prebiotics as an adjunctive treatment in schizophrenia, and was based on animal studies that explored two aspects of schizophrenia: cognitive dysfunction and antipsychotic-mediated weight gain.

There is strong evidence that schizophrenia and its treatment are associated with impaired cognitive functions. Schizophrenia subjects perform 1.5–2.0 standard deviations below healthy controls in a number of neurocognitive tasks, with largest impairments observed in working memory, attention, problem solving, processing speed, and social cognition (61). Particularly alarming are studies showing that antipsychotic drugs can induce cognitive impairments—risperidone was found to decrease spatial working memory despite clinical improvement (62), while lowering the dose of risperidone or olanzapine was found to significantly improve RBANS and DIEPSS scores (63). These changes can have significant functional implications such as poor work performance, hence addressing them is important in effective treatment.

Ingestion of the B-GOS® prebiotic formulation was found to significantly improve cognitive flexibility in rats (64). Although reduced cognitive flexibility is not unique to schizophrenia, B-GOS® supplementation in this disorder improved global cognitive performance measured by the Brief Assessment of Cognition in Schizophrenia [BACS, (61)], compared to placebo (65). This clearly requires replication with a larger study which should also include assessments of positive and negative symptoms so that the full therapeutic potential of prebiotics in schizophrenia is understood.

Although the mechanisms underlying the pro-cognitive effect of B-GOS® in schizophrenia are not clear, prebiotic supplementation in rats was also found to increase responses of PFC pyramidal neurones to the application of NMDA, and elevate cortical expression of GluN2B and GluN2A NMDA receptor subunits (64, 66). Furthermore, elevated hippocampal levels of BNDF have been reported (66). These changes are highly pertinent to schizophrenia, as NDMA hypofunction and decreased BDNF levels are thought to be involved in its pathogenesis, and its associated cognitive impairment (67).

## The Problem of Obesity in Schizophrenia, and the Potential Benefits of Prebiotics

Antipsychotic treatment is associated with increased body weight, and it is thought to be a key reason for higher rates of obesity among people with schizophrenia, along with lifestyle and social factors (68). This was robustly demonstrated in a meta-analysis of studies investigating this phenomenon, with clozapine and olanzapine increasing weight by >4 kg after 10 weeks of treatment (69). Furthermore, second-generation antipsychotics were found to induce extreme weight gain (≥7% body weight gain) in 7.7–17% of patients within the first year of treatment (70).

The relationship between obesity and schizophrenia is complicated and deserves further attention when discussing the role of the microbiome in this disorder. Obesity is known to be associated with altered gut microbiota, with most convincing evidence showing that transfer of “obese” microbiota to GF mice is associated with weight gain (71). However, the precise nature of those alterations is unclear, much like those in schizophrenia. Some studies identified a decrease in the abundance of *Bacteroidetes* and increase in *Firmicutes* [reviewed in (72)], which is interestingly a pattern also reported following olanzapine treatment (as discussed above)—a drug known to induce weight-gain. However, as there is such inconsistency among studies on the microbiomes in both conditions, it is impossible to make useful comparisons between them. Interestingly, there is evidence that the pathways discussed above as potentially affected by the microbiome in schizophrenia might also be affected in obesity. This pertains primarily to the inflammatory hypotheses of schizophrenia, as obesity is known to induce a chronic low-grade inflammatory state, with elevated levels of cytokines including IL-6 and TNF-α [reviewed extensively in (73)]. The parallels extend, however, further. Mutations in the BDNF gene have been found to be associated with hyperphagia and obesity in people (74, 75), while exogenous BDNF administration was shown to induce weight loss in animal models (76), although studies on serum BDNF levels in obese individuals have yielded ambiguous results (77). Likewise, metabolomic studies have shown elevated levels of kynurenate associated with high BMI in both adults (78) and children (79).

Unfortunately, interpreting the complicated relationship between schizophrenia, obesity, and the microbiome is made almost impossible by the lack of data indicating causal relationships. Perhaps surprisingly, two studies found a negative correlation between body weight and symptom severity in schizophrenia (80, 81), but this was likely an artifact of the effects of atypical antipsychotics on both weight and symptoms. On its own, however, obesity was found to be associated with lower cognitive function, although it is unclear which is antecedent, and whether obesity triggers specific mechanisms affecting cognition (82). Thus, it is unknown whether obesity could have a direct impact on symptoms of schizophrenia, or whether it is merely a side-effect of treatment or an associated morbidity with shared pathophysiological aspects, like BDNF alterations and inflammation. Likewise, there is no clear indication whether the changes in BDNF and kynurenate levels are a contributing cause or a consequence of obesity, whether they are of clinical significance, and whether the microbiome has any impact on them. Nonetheless, considering the importance of obesity in schizophrenia, it would be useful to see detailed studies analyzing the impact of obesity on specific pathophysiological correlates of this disorder, as well as methodologically consistent studies comparing microbiome profiles between schizophrenia and obesity, searching for common signatures.

Regardless of the impact of obesity on the pathophysiology of schizophrenia, the problem of obesity in the disorder needs to be addressed due to its medical implications, and prebiotics could prove useful for this purpose. The administration of B-GOS® reduced olanzapine-induced weight gain in rats (65), whereas in a mouse model of metabolic syndrome, an oligofructose prebiotic caused a trend decrease in weight gain, reductions in food and liquid intake and improved glucose tolerance (83). Prebiotic metabolism by gut bacteria produces short-chain fatty acids (SCFAs), which are agonists of G-protein coupled receptors GPR41 and GPR43 (84). These receptors are expressed in adipocytes, inhibit lipolysis and reduce plasma levels of free fatty acids (85). This could provide a potential mechanism for prebiotic-induced weight normalization and protection against metabolic syndrome, and is also consistent with reduced SCFA-producing bacteria reported in one study in subjects with schizophrenia (17). However, the metabolic effects of the B-GOS® prebiotic in rats were not observed in schizophrenia subjects following a 12 week supplementation (65). In this instance it is noteworthy that in rats the B-GOS® was administered 7 days prior to antipsychotic, whereas the schizophrenia patients had been on medications for several months or years prior to B-GOS® intake. Perhaps prebiotic supplementation would have more beneficial effects of started in parallel with antipsychotic prescription.

## Probiotics in Schizophrenia

Probiotics contain living beneficial bacteria, typically from genera *Lactobacilli* and *Bifidobacteria* (86). A randomized, placebo-controlled trial of a combination of *Lactobacillus rhamnosus* and *Bifidobacterium lactis* Bb12 in schizophrenia did not change PANSS scores over the course of the 14 week trial (87), though a trend increase in plasma BDNF was observed (88). This effect was also reported in a separate trial of *Bifidobacterium breve* in rats (89). The treated patients also reported less severe bowel movement difficulties, an important observation considering that constipation is a common side-effect of antipsychotic treatment (90) and can be fatal (91).

Recently, a probiotic supplement containing *Lactobacilli* and *Bifidobacterium bifidum* was given with vitamin D to schizophrenia subjects, which resulted in a significant improvement in the general and total PANSS scores, decreased circulating CRP levels and enhanced total antioxidant capacity of plasma, indicating symptomatic improvement and reduced inflammation (92). However, it is uncertain which component of the intervention was responsible for those changes. In another study of schizophrenia, consumption of *Bifidobacterium breve* A-1 for 4 weeks improved PANSS and anxiety/depression scores, increased levels of IFN-γ, IL-1R1, IL-10, IL-22, and decreased levels of TNF-α (93). The psychological effects are significant as the prevalence of depression and panic disorder in schizophrenia patients is 50 and 15% respectively (93). The increase in IL-22 is perplexing, as IL-22 is associated with both inflammatory reactions, and maintenance of the intestinal barrier and host responses against intestinal bacterial pathogens (94). The increased expression of IL-10 and decreased expression of TNF-α may further suggest anti-inflammatory effects, but is countered by elevated pro-inflammatory TNF-β and IL-1R1 (95, 96).

Taken together, the results of probiotic trials are highly discrepant, which could reflect differences in the treatments used. There is, however, preliminary evidence that probiotic supplementation could benefit people with schizophrenia both in terms of symptoms and co-morbid conditions, despite the apparent lack of effect on core aspects of the disorder. What is truly needed are larger-scale studies with consistency in treatment choices and measured outcomes.

## Conclusion

**Figure 1:**
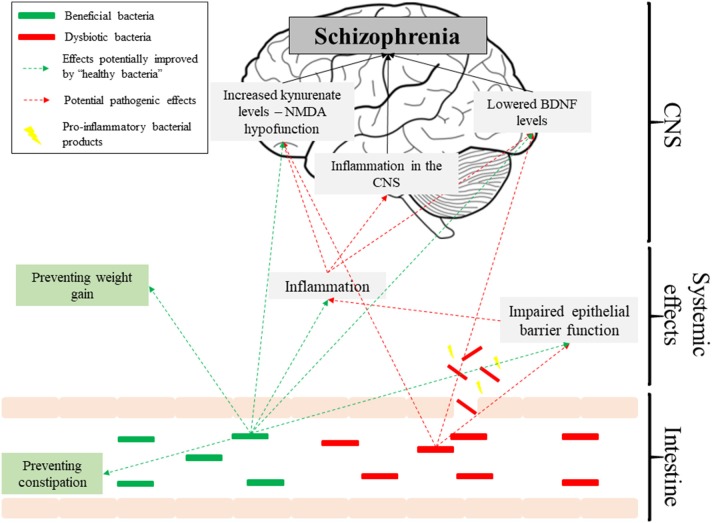
Pathways potentially affected by the microbiome in schizophrenia.

The existing evidence for dysbiosis as a factor in the pathogenesis of schizophrenia is underwhelming. None of the available studies are prospective, making causal relationships unfeasible, and the data is plagued with methodological inconsistencies. However, if future studies confirm that dysbiosis predicts schizophrenia, then it could link a number of observations in schizophrenia patients, such as raised inflammatory markers or altered BDNF and kynurenate levels, and thus contribute to the increasingly complex picture of its etiology (Figure 1). Despite limited evidence, there is promise in the use of pre/probiotics as auxiliary treatments in schizophrenia, aimed at improving side-effects of antipsychotics and complementing their action, particularly in terms of cognitive impairments. Overall, we should look with anticipation toward new studies published in this field.

## Author Contributions

TS and PB made an equal contribution to the conceptualization and drafting of the manuscript. TS drafted the final version of the document and constructed the accompanying figure and table. BL provided a significant contribution to clinical content and proofreading. AL and PB made equal contributions to scientific content proofreading.

### Conflict of Interest

The authors declare that the research was conducted in the absence of any commercial or financial relationships that could be construed as a potential conflict of interest.

## Abbreviations

GAF: global assessment of functioning
GF mice: germ-free mice
VTA: ventral tegmental area
LBP: LPS-binding protein
FMT: fecal microbiota transplant
PFC: pre-frontal cortex
BDNF: brain-derived neurotrophic factor
RBANS: repeatable battery for assessment of neuropsychological status
DIEPSS: drug-induced extrapyramidal symptoms scale
PANSS: positive and negative syndrome scale
GWAS: genome wide association study
TrkB: tropomyosin receptor kinase B (the BDNF receptor)
hs-CRP: high-sensitivity C-reactive protein.
